# Enhanced exchange bias and improved ferromagnetic properties in Permalloy–BiFe_0.95_Co_0.05_O_3_ core–shell nanostructures

**DOI:** 10.1038/srep18203

**Published:** 2015-12-14

**Authors:** K. Javed, W. J. Li, S. S. Ali, D. W. Shi, U. Khan, S. Riaz, X. F. Han

**Affiliations:** 1 Beijing National Laboratory for Condensed Matter Physics, Institute of Physics, Chinese Academy of Sciences, Beijing 100190, China; 2 Department of Electrical Engineering, CIIT WAH, Pakistan; 3 Centre of Excellence in Solid State Physics, University of the Punjab, Lahore-54590, Pakistan

## Abstract

Hybrid core–shell nanostructures consisting of permalloy (Ni_80_Fe_20_) and multiferroic(BiFeO_3_, BFO/BiFe_0.95_Co_0.05_O_3_, BFC) materials were synthesized by a two-step method, based on wet chemical impregnation and subsequent electrodeposition within porous alumina membranes. Structural and magnetic characterizations have been done to investigate doping effect on magnetic properties and exchange bias. The magnetometry analysis revealed significant enhancements of the exchange bias and coercivity in NiFe-BFC core-shell nanostructures as compared with NiFe-BFO core-shell nanostructures. The enhancements can be attributed to the effective reduction of ferromagnet domain sizes between adjacent layers of core-shell structure. It indicates that it is possible to improve properties of multiferroic composites by site-engineering method. Our approach opens a pathway to obtain optimized nanostructured multiferroic composites exhibiting tunable magnetic properties.

One dimensional (1D) nanostructures such as nanotubes (NTs) and nanowires (NWs) attract considerable scientific attention because of their novel structure, physical properties, and potential applications in ultrahigh magnetic recording media, ultra-small magnetic sensors, drug delivery etc.^1^,^2^ which can differ significantly from their bulk or thin film counter analogues^3^,^4^,^5^. Multiferroics show simultaneous ferroelectric and magnetic orders, and are promising materials for design and synthesis of multifunctional devices^6^. It has been proven that the magnetic and ferroelectric order can coexist in some unusual perovskite type oxide materials, termed as multiferroics^7^,^8^,^9^,^10^. Among them, BiFeO_3_ (BFO) has received the most attention^11^,^12^,^13^,^14^. Since nanostructures might possess excellent properties, it is worth to combine BFO with 1D nanostructures. Coaxial nanostructures, which may exhibit additional effects and have potential applications as multi-functional materials, have attracted considerable attention both for fundamental and technological interest^15^,^16^,^17^,^18^. Thin films consisting of ferromagnetic materials and BFO have been widely studied in recent years^19^,^20^.

Problems of small ferromagnetism^21^, high leakage current^22^, and appearance of secondary phases^23^ have hindered the practical application of BFO to devices. To overcome these problems, much effort has been devoted, such as rapid liquid phase sintering^24^, sintering followed by quenching or leaching^23^, chemical substitution^25^ and epitaxial strain^26^. However, the getting of both large magnetization and high resistance in BFO remains to be the major obstacle for its potential applications. In the past few years, site-engineering technique by substitution of small amount of impurities was proposed to solve this problem, and reduced leakage current properties were reported^27^,^28^,^29^,^30^,^31^. To make samples show large magnetic moment that may produce large magnetoelectric coupling and then make BFO possible to be used in industries, ion substitution is an effective method. In literature it is pointed out that Co^2+^ substitution for Fe^3+^ could improve the magnetic property of BFO ceramics^32^. The same phenomenon also occurred in the BFO thin film^28^. Much effort has been paid to improve the magnetization in BFO to get a sizable response to the application of magnetic field. Most of the work concentrated on the Bi site ion substitution, for example, the equivalent hydrostatic pressure by Ca doping increases the magnetic Neel temperature has been studied by Catalan *et al.*^33^. Fe-site ion substitution with magnetic ions is also very attracting, and enhanced ferromagnetism was reported^32^. During last decade, we have already reported several studies on 1D ferromagnetic nanostructures both elemental (Co, Ni, Fe) as well as their alloys^34^,^35^. The use of multiferroic structures in spintronics is a rapidly developing field of interest, and a number of possible device architectures have been proposed^36^.

In this work, we prepared the hybrid permalloy–multiferroic core–shell nanostructures. Permalloy (Ni_80_Fe_20_) and bismuth ferrite (un-doped and Co doped) served as the core and shell of the hybrid core-shell nanostructures, respectively. We have investigated the magnetic properties and the magnetic reversal mechanism of these core–shell nanostructures as well. Furthermore, we have discussed the exchange bias effect (*H*_*ex*_), which results from the exchange coupling between the ferromagnetic core and the antiferromagnetic BFO shell. These combined ferroelectric and antiferromagnetic functionalities of BFO are important for future multiferroic devices.

## Results

**Figure 1:**
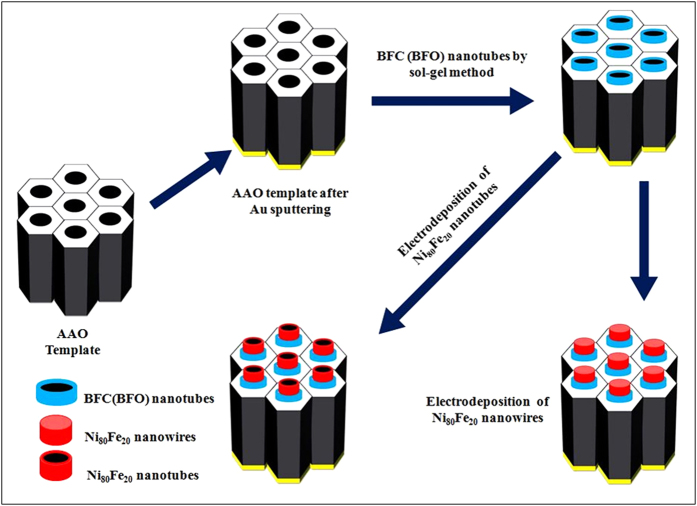
Ni_80_Fe_20_–BFC(BFO) core–shell nanostructures prepared by template assisted sol–gel and electrodepostion by two step method.

**Figure 2:**
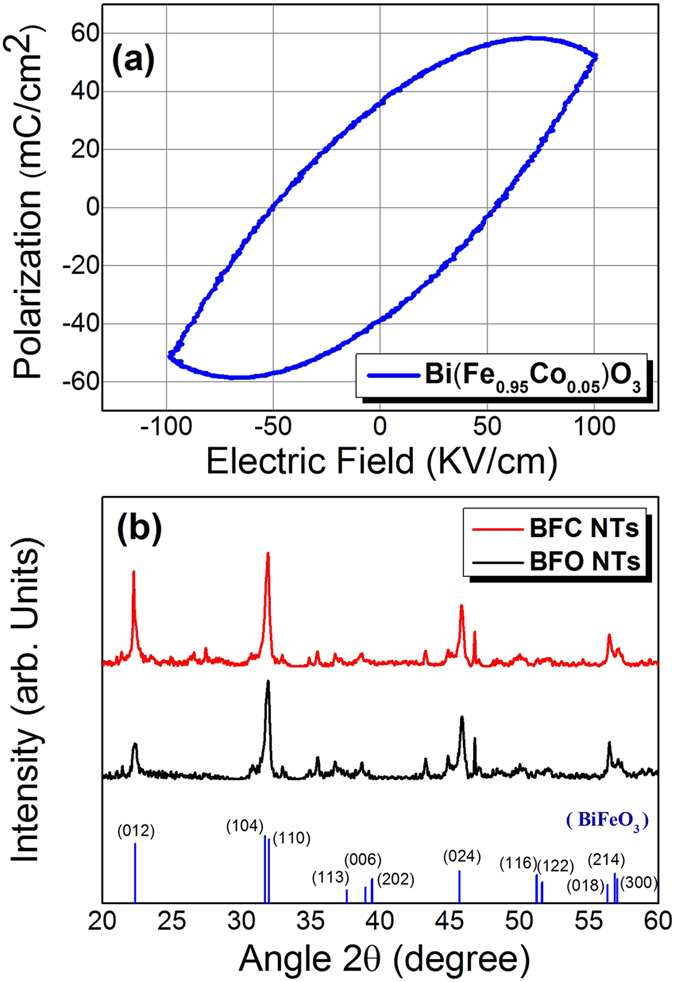
(**a**) The ferroelectric bipolar P-E hysteresis loop of the BFC (BiFe_0.95_Co_0.05_O_3_) nanotubes arrays. (**b**) XRD patterns for BFO (BiFeO_3_) and BFC (BiFe_0.95_Co_0.05_O_3_) nanotubes.

It is expected that with suitable doping better ferroelectric properties could be achieved in BFO. Figure 1 shows the schematic for synthesis process. The P–E hysteresis loops of Co doped BFO nanotubes samples indicate their ferroelectric nature but with loosy features, as shown in Fig. 2(a) (P-E loop for un-doped BFO has been shown in Figure S1†). To understand this behaviour, we have to first note that in BiFeO_3_, small amounts of Fe^2+^ ions and oxygen vacancies exist. Incidentally, BiFeO_3_ shows p-type conductivity^32^, which can be understood by considering the substitution of a small amount Fe^2+^ ions in Fe^3+^ positions (acceptor doping of Fe^3+^ by Fe^2+^). When Co^2+^ substitutes Fe^3+^, the acceptor doping of Fe^3+^ by Fe^2+^ is reduced and this causes a decrease in the conductivity. Hence, Co^2+^ doped BiFeO_3_ show comparatively less-loosy ferroelectric hysteresis loops compared to un-doped BiFeO_3_^37^.

**Figure 3:**
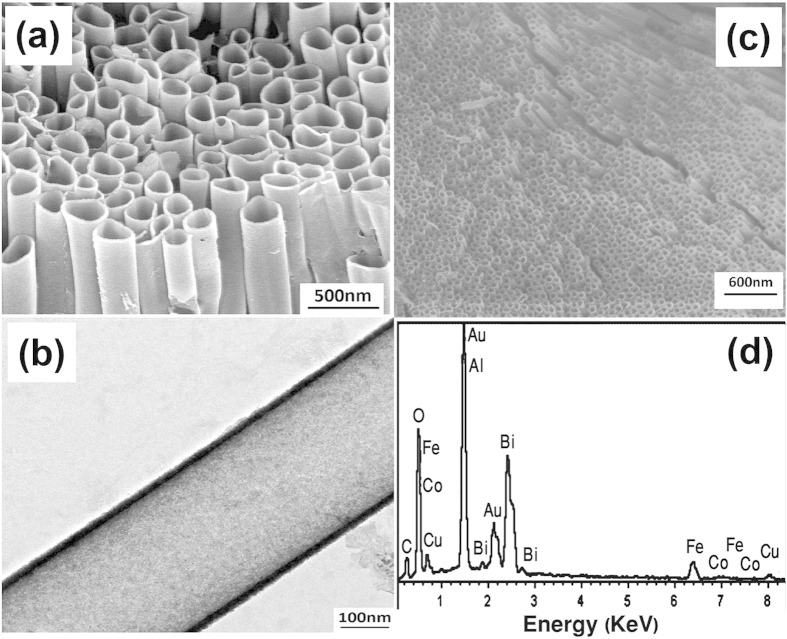
SEM image of BFC nanotubes with a diameter of (a) 300 nm (c) 100 nm. (**b**) TEM image of a single BFC nanotube with 300 nm. (**d**) Composition of the BFC nanotubes obtained from the EDS.

Figure 2(b) shows the XRD analysis for BFO (BiFeO_3_) and BFC (BiFe_0.95_Co_0.05_O_3_) nanotubes. BFC exhibited the similar rhombohedral perovskite structure R3c as BFO. XRD analysis showed that no impurity phase such as cobalt oxide was detected. But energy dispersive X-ray (EDS) pattern clearly demonstrated the existence of Co, as shown in Fig. 3(d). Also, It is reported that magnetization decreases when the addition of Co is further increased to x >0.07, suggesting that the ferromagnetism of BiFe_1−x_Co_x_O_3_ does not increase with the increase of Co dopant. It indicated that there is no impurity phase such as cobalt oxides in the as-prepared samples; otherwise, there should be an increased ferromagnetism with the increased Co dopant which conflicts with the above results^38^. Therefore, it is reasonable to consider that doped Co ions have been effectively incorporated into the crystal structure of BiFeO_3_.

In this report, alumina membranes with two different pore diameters of 100 nm and 300 nm were used for the synthesis of NiFe–BFO and NiFe-BFC core–shell nanostructures. AAO templates with smaller diameters were prepared by two-step anodization method whereas for larger diameter we have used whatman membranes. Core-shell nanostructures were prepared by two-step method. First BFO/BFC nanotubes were synthesized with the help of sol-gel method. The single phase perovskite structure of BFO/BFC shell with least secondary phases was obtained by annealing at 650 °C. After that Ni_80_Fe_20_ NWs/NTs were electrodeposited. The synthesis of phase pure and stoichiometric bismuth ferrite is challenging mainly because of the volatility of bismuth and the other more stable competing phases. Furthermore, the annealing temperature was optimized to obtain a single phase perovskite crystal structure. The templates containing the precursors were annealed at 550 °C–700 °C and with 25 °C step. The nanotubes annealed at higher temperatures were found to be having the least secondary phases as compared with 600 °C. According to literature as well as in our previous studies^39^,^40^, it has been found that at temperature >650 °C, AAO template start interacting with nanowires, so to avoid any misguidance in results, we have done annealing at 650 °C. Also, at this temperature we found the least secondary phases like second phase Bi_2_Fe_4_O_9_. Figure 2(b) shows the XRD patterns of BFO and BFC nanotubes after the templates were dissolved.

Figure 3(a,c) shows the morphology of BFC nanotubes with different diameters, characterized by using a scanning electron microscope (SEM) and a transmission electron microscope (TEM). Many diffraction peaks due to the BFO structure was observed, indicating the formation of a polycrystalline structure. It has been observed that the (012) diffraction peak of the Co added BFO becomes stronger as compared with un-doped BFO. Similar kind of observations have also been reported for thin films^28^. The standard XRD pattern of BiFeO_3_ (space group R3c) was also shown for comparison. By adding an appropriate amount of excess bismuth and optimizing the annealing temperature, the unexpected second phase can be minimized.

BFO/BFC nanotubes were of polycrystalline structure. The composition of the BFC nanotubes has been checked by using the energy dispersive spectrum (EDS) as shown in Fig. 3(d). Similar morphology has also been observed for un-doped BFO nanotubes (Figure S2† & S3†). The magnetic properties of BFO/BFC nanotubes were investigated by using Vibrating sample magnetometer (VSM) at room temperature. Because BFO is antiferromagnetic at room temperature and the weak local canting moment being completely cancelled by the averaging effect, So no and/or extremely weak ferromagnetism was observed for BFO/BFC nanotubes.

**Figure 4:**
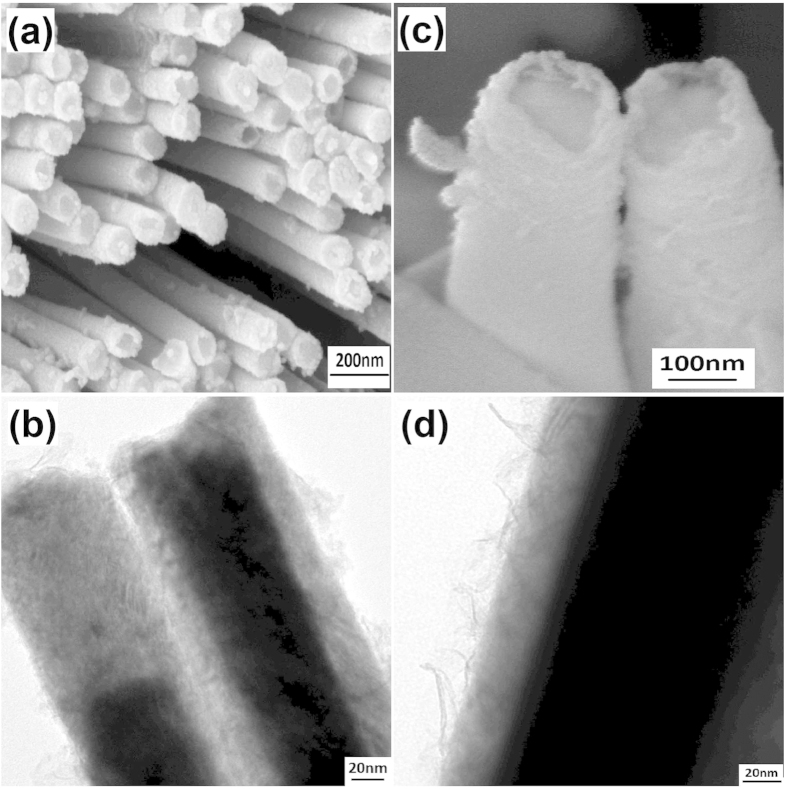
(**a,b**)SEM and TEM images of NiFe-BFC core-shell nanowires with diameter 100 nm. (**c,d**) SEM and TEM images of NiFe-BFC core-shell nanowires with diameter 300 nm, respectively.

The AAO templates with a pore size of 100 nm and 300 nm filled with BFO/BFC nanotubes were used for the electrodepostion of Ni_80_Fe_20_ nanowires. Figure 4 shows the SEM (a,c), and TEM (b,d) images of NiFe-BFC core-shell nanowires with 100 nm and 300 nm, respectively. We have observed that the influence of thin BFO/BFC shell that acts on Ni_80_Fe_20_ core nanowires with diameter 300 nm is extremely small or negligible. As core diameter (~274 nm) is very large as compared with shell thickness (~24 nm). So, NiFe–BFC(BFO) core–shell nanowires prepared by using diameter of 100 nm was selected for more detailed analysis. Both SEM and TEM images shows a fine core-shell structure. The AAO templates with a pore size of 300 nm filled with BFC/BFO nanotubes were used for the NiFe-BFC(BFO) core-shell nanotubes as well. The SEM and TEM images of NiFe-BFO coreshell NWs/NTs has also been shown in Figure S4†.

**Figure 5:**
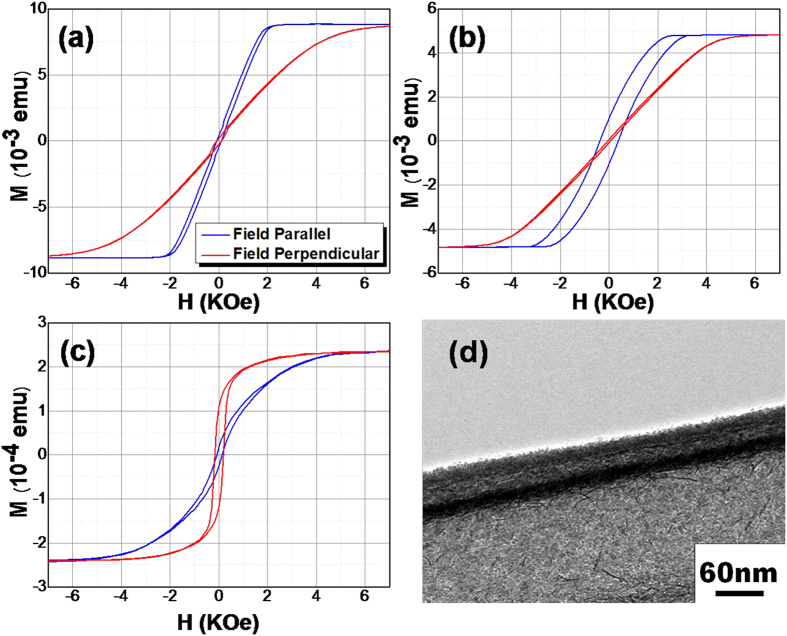
M-H curves for NiFe-BFC coreshell nanowires with diameter (**a**) 300 nm and (**b**) 100 nm. (**c**) M-H curves for NiFe-BFC core-shell nanotubes with diameter 300 nm (**d**) TEM of NiFe-BFC core-shell nanotube.

Figure 5a,b shows the M-H curves for NiFe-BFC nanowires with 300 nm and 100 nm diameter, respectively. Whereas, Fig. 5c,d shows the M-H curve and TEM image for NiFe-BFC core-shell nanotubes. The wall thickness of BFO/BFC nanotubes is about 22-25 nm, which is comparable with the thickness of electrodeposited Ni_80_Fe_20_ nanotubes. The hybrid multiferroic nanostructure (BTO–Co) reported by Narayanan *et al.*, demonstrates the existence of magnetoelectric coupling via magnetocapacitance measurements, indicating the possible tunability of the dielectric permittivity with an external magnetic field^18^. To make sure electrodeposited core is Ni_80_Fe_20_, XRD analysis for core-shell nanostructure has been done. As from EDS, it can’t be confirmed that the electrodeposited core consists of Ni_80_Fe_20_ and not only the Ni. XRD spectrum for BFO/BFC NTs and core-shell structure clearly shows that core is Ni_78_Fe_22_ (Figure S5†). The M-H curves for NiFe-BFO NWs/NTs have also been shown in Figure S6†.

**Figure 6:**
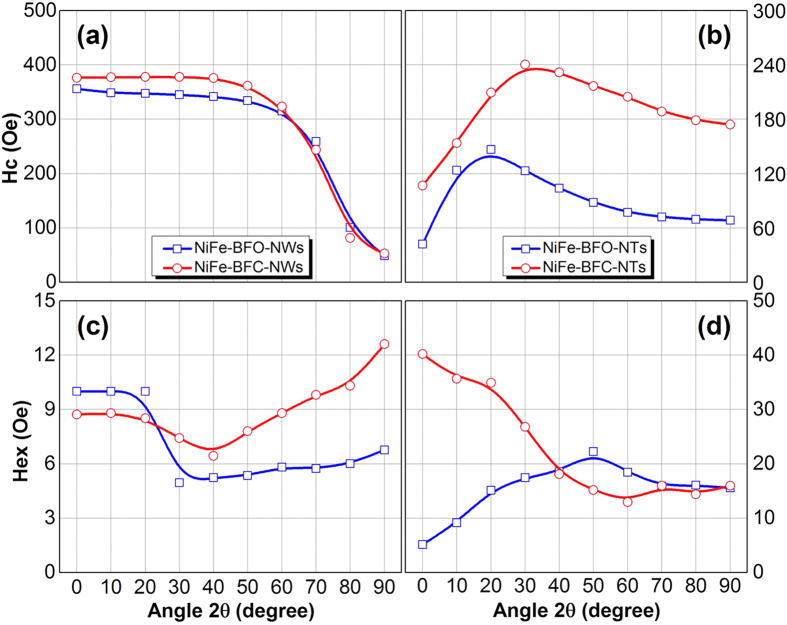
Angular dependence of (a,b) Coercivity *H*_*c*_ and (c,d) exchange bias field *H*_*ex*_ for NiFe-BFO and NiFe-BFC core-shell nanowires (NWs) and nanotubes (NTs).

In this report, a hybrid one-dimensional nanostructure with a ferromagnetic layer (permalloy) in contact with the multiferroic BFO as well as BFC layer were fabricated and the coupling between them was investigated. It has been reported that the antiferromagnetic order of BFO can be used to establish exchange bias in the hybrid nanostructures^19^, which leads to possible modification in ferromagnetic magnetization through the exchange bias effect. Magnetic properties of NiFe–BFC(BFO) core–shell NWs/NTs were investigated as shown in Fig. 6.

Figure 6a,c shows the angular dependence of coercivity *H*_*c*_ and exchange field *H*_*ex*_ for NiFe-BFC core–shell nanowires with a diameter 100 nm, at room temperature. The easy axis orients along the nanowires due to the strong shape anisotropy, also can be seen from Fig. 5(b). An obvious loop shift observed for both directions. The observation of enhanced coercivity *H*_*c*_ and exchange bias field *H*_*ex*_ can be attributed to the presence of uncompensated spin at the interface between permalloy core and antiferromagnetic (BFO/BFC) shell, which has been enhanced after doping BFO with Co. Both the *H*_*ex*_ and *H*_*c*_ was enhanced slightly for NiFe-BFC core-shell nanowires as compared with NiFe-BFO core-shell nanowires. It is to be noted that the variation almost remains the same for un-doped and doped BFO nanostructures. The coercivity decreases for both NiFe-BFO and NiFe-BFC core-shell nanowires with the increasing angle, which indicates the dominance of coherent rotation mode in the magnetization reversal process. The coercivity remains almost constant upto 40 degree for NiFe-BFC as compared with NiFe-BFO. For NiFe-BFO, *H*_*ex*_ is maximum for lower angles, then dropped sharply after 20 degree angle before rising slightly again. The behaviour is almost same in case of NiFe-BFC nanowires except the decrease upto 40 degree is gradual, and *H*_*ex*_ become maximum at 90 degree unlike NiFe-BFO where it was maximum at zero degree.

Figure 6b,d shows the angular dependence of coercivity *H*_*c*_ and exchange field *H*_*ex*_ for NiFe-BFC core-shell nanotubes with a diameter of 300 nm measured at room temperature. In case of NiFe-BFC(BFO) core-shell nanotubes the easy axis orients perpendicular to nanotubes axis as compared with core-shell nanowires, as shown in Fig. 5(c), also in agreement with literature^35^. For magnetic nanowires the magnetization reversal mechanism depends upon the diameter of the nanowires. The *H*_*ex*_ became maximum when the magnetic field was along the nanowires axis, but after doping with Co, *H*_*ex*_ field becomes maximum when magnetic field was perpendicular to the nanowires axis. It may be attributed to the change in spin structure at the interface and the microstructure of both the Ni_80_Fe_20_ nanowires and the BFO/BFC shell layer. The anisotropic field of the nanotubes is mainly determined by three contributions. First, the shape anisotropy field which tends to induce a magnetic easy axis parallel to the nanotubes axis; Second, the magnetostatic dipole interaction field which will induce a magnetic easy axis perpendicular to the nanotubes axis; Third, the magnetocrystalline anisotropy field. The magnetocrystalline anisotropy field can be neglected since the nanotubes fabricated by the electrodeposition method are usually poly-crystalline/amorphous.

Exchange Bias has been found in a variety of systems with FM/AFM interfaces, including core-shell nanoparticles, thin film systems, and other lithographed nanostructures. But, only a few reports were found on the EB coupling in core-shell nanoparticles, and nanotubular structures. When a bulk system is reduced to the nanoscale, the coupling effect at the FM/AFM interface can be drastically increased^41^. The domain size in AF was shrinked by Co doping which is related to the enhancement of the exchange bias effect. This decrease in domain size due to Co doping causes the spin rearrangement and hence the exchange coupling has been enhanced. So, both coercivity *H*_*c*_ and exchange bias field *H*_*ex*_ were increased after doping. This exchange bias at the interface leads to a preferred orientation of magnetic moment in ferromagnetic layer, enhancement of coercivity *H*_*c*_, and a shifted hysteresis loop from the origin. There are several parameters such as interfacial spin configuration, interface roughness, crystalline grain size and AF layer thickness which affect the exchange bias mechanism.

The origin of unidirectional anisotropy and exchange bias in the AFM-FM hetero-structures is assumed to be an exchange interaction at the interface. An enhanced *H*_*c*_ has been observed for doped BFO nanostructures whereas magnetization reversal mechanism was the same i.e. from curling to coherent rotation. An improved *H*_*c*_ as well as an enhanced *H*_*ex*_ was observed for NiFe-BFC core-shell nanotubes, which originate from exchange bias coupling. One possible reason for such behavior is the rearrangement of antiferromagnetic domains during the electrodeposition of ferromagnetic nanotubes inside the shell^19^. It has been noted that *H*_*ex*_ was increased from minimum upto ≤40 degree for NiFe-BFO core-shell nanotubes, whereas it decreased from maximum upto ≤40 for NiFe-BFC core-shell nanotubes. Above 40 degree behaviour remained almost same for both un-doped and doped core-shell nanotubes. It is to be noted that the *H*_*ex*_ enhanced significantly after Co doping for core-shell nanotubes as compared with core-shell nanowires, as shown in Fig. 6(c,d). It may be attributed to larger core diameter compared with BFO/BFC shell thickness in core-shell nanowires, whereas wall thickness was very comparative in case of core-shell nanotubes. The measurements showed the enhanced ferromagnetism for the sample with Co doping. These enhanced properties of the Co doped BFO nanotubes may be mainly attributed to the lattice distortion and the breakage of the spin cycloid period of the BFC nanotubes, respectively^42^.

Mainly there are three types of reversal modes (coherent, curling and nucleation) to investigate the angular dependence of coercivity. In general, magnetization reversal mechanism depends on the diameter of 1D nanostructure. The critical diameter for transition from coherent to non-coherent rotation is


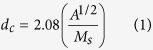


Where *A* is exchange stiffness and *M*_*s*_ is the saturation magnetization^43^. For NWs/NTs having diameter larger than *d*_*c*_, the magnetization reversal mechanism can be explained by curling mode, and coercivity *H*_*c*_ decreased with increase in diameter. The relation between coercivity *H*_*c*_ and critical diameter *d*_*c*_ has already been discussed in detail for nanowires^44^.

As for core-shell nanotubes we have used diameter 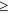
200 nm, which is much greater than *d*_*c*_ for coherent rotation, reversal is expected by curling mode. The curling mode of reversal for 1D nanostructure shows that.


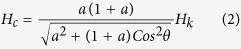


Where


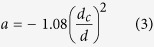


It shows that Coercivity increases as the angle increases whereas Coercivity decreases as the angle increases for coherent rotation^45^,^46^. It is worth noting that coherent mode is only suitable for shorter nanotubes, whereas, in present study the length of nanotubes was in the range of a few micrometers. So, a transition from curling to coherent mode for the NiFe–BFC(BFO) core–shell nanotubes with the increasing angle was used to understand the magnetization reversal mechanism. Since the diameter of the nanotubes is much larger than the critical diameter, coercivity increases with the increase of angle according to the curling mode for smaller angles, whereas at larger angles, magnetization reversal will take place by coherent rotation. The curves with M-type nature (Fig. 6b) illustrate that curling occurred at smaller angles while coherent rotation is dominant for lager angles. These results are in agreement with literature and our previous studies^35^. It has been observed that the critical angle for magnetization reversal slightly increased from 30 degree to 40 degree after Co doping.

Now, for our core-shell nanowires Coercivity *H*_*c*_ decreases for both cases, as shown in Fig. 6(a), core-shell nanowires with the increasing angle, which indicates the dominance of coherent rotation mode in the magnetization reversal process. And for core-shell nanotubes (Fig. 6(c)), Coercivity increased with angle initially but above the critical angle it started to decrease, showing M-type variation, which illustrates that coherent rotation is dominant at larger angles and curling happens for smaller angles. So, we can say that the magnetic moments will align parallel to nanotubes axis for smaller angles, so reversal happens by curling mode of rotation. Whereas, these moments will align perpendicular to the nanotubes axis for larger angles and coherent reversal mode will be observed.

**Figure 7:**
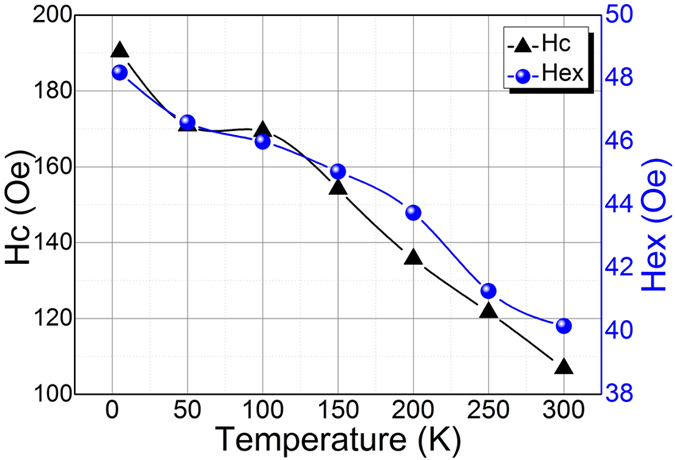
Coercivity (*H*_*c*_) and Exchange bias field (*H*_*ex*_) temperature dependence for NiFe-BFC nanotubes.

The wall thickness of the nanotubes can strongly affect the mechanism of magnetization reversal and the overall magnetic behaviour, while for magnetic nanowires the magnetization reversal mechanism depends upon the diameter of the nanowires. Shorter the diameter, higher will be the exchange bias. The *H*_*ex*_ reaches to maximum when the magnetic field was along the hard axis for both Co doped core-shell nanowires as well as nanotubes, unlike NiFe-BFO core-shell nanowires where *H*_*ex*_ reaches to maximum when magnetic field was along easy axis. There are many factors e.g. magnetic anisotropy, interface roughness, spin configuration and magnetic/antiferromagnetic domains which may affect the exchange interaction, so, these results may be attributed to different crystalline orientations in the microstructure of Ni_80_Fe_20_ nanotubes walls and the BFO/BFC shell. The temperature dependence of Coercivity *H*_*c*_ and *H*_*ex*_ was shown in Fig. 7. The small increase in *H*_*c*_ for lower temperatures can be explained by thermal activation over the energy barrier. The comparatively sharper rise below 50 degree can be attributed to the large surface/volume ratio and the existence of super-paramagnetic nanoparticles^47^.

## Discussion

Upto now, most studies have been done on thin films consisting of ferromagnetic materials and BFO. Small ferromagnetism, high leakage current, and appearance of secondary phases have hindered the practical application of BFO to devices. During last few years, site-engineering has proven to be a promising method to enhance and improve properties of such nanostructures. Most of the work concentrated on the Bi site ion substitution. In our previous work, a detailed study on the synthesis of Ni-BFO nanostructures was presented. Characterizations on the ferroelectric and magnetic properties confirmed the coexistence of spontaneous electric polarization and antiferromagnetic spin ordering in the BFO nanomaterials. We had experimentally demonstrated that ferromagnetic cores are exchange-coupled to the multiferroic BFO shell. Small but evident exchange bias was found in the Ni–BFO core–shell nanostructures without any magnetic field annealing process^48^.

In this report we studied the Fe site doping effect on magnetization and exchange bias field for core-shell nanostructures. A detailed study on the synthesis of Co doped and un-doped bismuth ferrite core-shell nanostructures have been done. We demonstrated that, by optimizing the sol and the heat treatment conditions, it is possible to grow well ordered BFO/BFC nanotubes with the diameter 100 nm and 300 nm. Analysis confirmed the co-existence of spontaneous electric polarization and antiferromagnetic spin ordering in the BFO/BFC nanotubes. In addition, the deposited ferromagnetic NWs/NTs lead to the formation of ferromagnetic–multiferroic core–shell nanostructures. By doping Co in BFO and using NiFe as core (compared with Ni-BFO) a significantly enhanced *H*_*ex*_ and improved *H*_*c*_ has been observed.

In summary, significantly enhanced exchange bias field *H*_*ex*_ was observed in case of NiFe-BFC core-shell nanotubes. It is attributed to the rearrangement of the anti-ferromagnetic domains during the electro-deposition of Ni_80_Fe_20_ NWs/NTs inside the BFO/BFC shell. Thus, we have demonstrated that ferromagnetic cores are exchange-coupled to the multiferroic BFO/BFC shell, which can be enhanced by doping appropriate material. So, ferromagnetic/ferroelectric 1D nanostructures with electric-field control of magnetization or magnetic-field control of polarization may be realized. The synthetic methods presented in this work can be easily expanded to the fabrication of other similar doped hybrid core-shell nanostructures.

## Methods

The chemical solutions for BiFeO_3_ (BFO) and BiFe_0.95_Co_0.05_O_3_ (BFC) were prepared by dissolving bismuth nitrate [Bi(NO_3_)_3_.5H_2_O], iron nitrate [Fe(NO_3_)_3_.9H_2_O] and cobalt nitrate [Co(NO_3_)_2_.6H_2_O] in ethylene glycol [C_3_H_8_O_2_] to form the final products, and were used without any further purification (All chemical salts were purchased from Sinopharm Chemical Reagent Beijing Co., Ltd.). For templates with a diameter of 300 nm were bought from Whatman International Ltd., and AAO templates with a diameter of 100 nm were obtained by a two-step electrochemical anodization process of aluminium sheet.

Both BFO and BFC nanotubes were fabricated by sol-gel method. The sol-gel precursor were optimized and prepared as follows: high purity Bi(NO_3_)_3_·5H_2_O and Fe(NO_3_)_3_·9H_2_O with a molar ratio of 1.1:1 (for BFO) and 1.1:0.95:0.05 (for BFC) were dissolved in C_3_H_8_O_2_. 10 mol % of excess Bi was added to compensate the bismuth loss during the heat treatment. The pH of the sol solution was adjusted by adding appropriate amount of nitric acid. The sol was stirred for 1 hour at room temperature. AAO templates with two different pore sizes: 300 nm and 100 nm were then dipped into the sol for 20 min. Templates containing the precursors were annealed at 650 °C, resulting in the formation of nanotubes in the pores of the AAO templates^48^.

The morphologies of the nanostructures were characterized by scanning electron microscope (SEM; Hitachi S-4800) and Transmission electron microscope (TEM; JEOL 2011). Both the SEM and TEM analysis were performed after dissolving the AAO template in 1 M NaOH solution. For TEM analysis, the AAO templates were completely dissolved in 1 M NaOH for 12 h at 60 °C. The structural analysis of the BFC/BFC nanotubes was obtained from XRD (RIGAKU; D/MAX-2400) results. The ferroelectric characteristic of nanotubes was studied by ferroelectric tester (Radiant; Premier II). Magnetic hysteresis curves (M-H) of the NiFe-BFC(BFO) core-shell nanostructures were measured by VSM (Microsense EV-9), whereas low temperature measurements were carried out using superconducting quantum interference device (SQUID).

## Additional Information

**How to cite this article**: Javed, K. *et al.* Enhanced exchange bias and improved ferromagnetic properties in Permalloy-BiFe_0.95_Co_0.05_O_3_ core-shell nanostructures. *Sci. Rep.* **5**, 18203; doi: 10.1038/srep18203 (2015).
